# lncRNA SSTR5-AS1 Predicts Poor Prognosis and Contributes to the Progression of Esophageal Cancer

**DOI:** 10.1155/2023/5025868

**Published:** 2023-01-23

**Authors:** Yuhao Hu, Ning Mao, Wei Zheng, Bin Hong, Xiong Deng

**Affiliations:** Cardiothoracic Surgery, Yongchuan Hospital of Chongqing Medical University, Chongqing, China

## Abstract

Esophageal cancer (ESCA), as a common cancer worldwide, is a main cause of cancer-related mortality. Long noncoding RNAs (lncRNAs) have been shown in an increasing number of studies to be capable of playing an important regulatory function in human malignancies. Our study is aimed at delving into the prognostic value and potential function of lncRNA SSTR5-AS1 (SSTR5-AS1) in ESCA. The gene expression data of 182 ESCA samples from TCGA and 653 nontumor specimens from GTEx. The expressions of SSTR5-AS1 were analyzed. We investigated whether there was a correlation between the expression of SSTR5-AS1 and the clinical aspects of ESCA. In order to compare survival curves, the Kaplan-Meier method together with the log-rank test was utilized. The univariate and multivariate Cox regression models were used to analyze the data in order to determine the SSTR5-AS1 expression's significance as a prognostic factor in ESCA patients. In order to investigate the level of SSTR5-AS1 expression in ESCA cells, RT-PCR was utilized. CCK-8 trials served as a model for the loss-of-function tests. In this study, we found that the expressions of SSTR5-AS1 were increased in ESCA specimens compared with nontumor specimens. According to the ROC assays, high SSTR5-AS1 expression had an AUC value of 0.7812 (95% CI: 0.7406 to 0.8217) for ESCA. Patients who had a high level of SSTR5-AS1 expression had a lower overall survival rate than those who had a low level of SSTR5-AS1 expression. In addition, multivariate analysis suggested that SSTR5-AS1 was an independent predictor of overall survival for ESCA patients. Moreover, RT-PCR experiments indicated that SSTR5-AS1 expression was distinctly increased in three ESCA cells compared with HET1A cells. CCK-8 experiments indicated that silence of SSTR5-AS1 distinctly inhibited the proliferation of ESCA cells. Overall, ESCA patients with elevated SSTR5-AS1 had a worse chance of survival, suggesting it could be used as a prognostic and diagnostic biomarker for ESCA.

## 1. Introduction

More than 300,000 people are diagnosed with esophageal cancer (ESCA) every year, making it one of the most common malignant tumors of the upper gastrointestinal tract and a serious threat to human health [1, 2]. Recent researches have indicated that a combination of factors, such as unhealthy lifestyle choices, exposure to carcinogens, underlying disorders, and genetics, has a role in the development of esophageal cancer [3, 4]. There are a number of risk factors that can enhance an individual's susceptibility to ESCA. These include an excessive consumption of mold, a genetic predisposition, an addiction to nicotine or alcohol, and a high intake of nitrosamines [5, 6]. At the present time, patients diagnosed with ECCA typically undergo one or more of the following standard treatment modalities: chemotherapy, radiation therapy, or surgery [7, 8]. Despite recent advancements in clinical interventions, the clinical outcomes for ESCA remain dismal, with <20% of patients surviving for five years after diagnosis [9, 10]. The late diagnosis, high frequency of metastases, and rapid progression of the tumor all contribute to the dismal survival rate of ESCA patients [11]. In addition, the specific genetics as well as the molecular pathways that are involved in ESCA are not well understood. The TNM stage is currently the most important factor in determining the prognosis of ESCA patients [12]. TNM stage is helpful; however, it can be different for different people even when the cancer is at the same stage [13]. In addition, clinicopathological characteristics include molecular comprehensive reflections, which include genes and proteins [14]. Thus, it is of the utmost need to create a reliable predictive biomarker that can predict the clinical prognosis of ESCA patients as quickly as possible.

Only 3% of the human genome is transcribed into protein-coding mRNAs, but around 75% of the human genome is translated into RNA [15]. In the past, ncRNAs were considered to be “evolutionary garbage” because it was believed that they did not have any biological roles [16, 17]. However, as a result of advances in deep sequencing technology, there is mounting evidence to suggest that noncoding RNAs (ncRNAs) have a significant influence on the molecular mechanisms of animals and even humans. Long noncoding RNA (lncRNA) refers to a set of transcripts of the noncoding RNA class that are longer than 200 nucleotides but do not have the ability to translate proteins into other forms [18]. There is evidence that long noncoding RNAs, or lncRNAs, play regulatory functions in a variety of biological processes, such as the differentiation of cells, the maintenance of stem cells, and the regulation of epigenetics [19, 20]. An imbalance in the expression of lncRNAs has been linked to a variety of human diseases, including cancer, neurological disorders, and cardiovascular conditions [21–23]. For instance, Wu et al. reported that higher expressions of DLEU2 or low levels of miR-30a-5p expression were an independent predictive predictor of poor survivals and tumor recurrences in lung cancer, and both were observed in non-small-cell lung cancer specimens. DLEU2 knockdown inhibited carcinogenesis and lung cancer invasion via targeting miR-30a-5p [24]. Xu et al. showed that the level of KCNMB2-AS1 was dramatically increased in ESCA. Through the modulation of the miRNA-3194/PYGL axis, the downregulation of KCNMB2-AS1 was able to inhibit stemness as well as proliferation, invasion, and migration [25]. Although research on lncRNAs is fairly restricted at the moment, the roles that lncRNAs play are incredibly diverse and comprehensive.

In this study, we evaluated TCGA datasets and discovered a novel lncRNA connected to ESCA called SSTR5-AS1. Patients with ESCA showed a high level of SSTR5-AS1. After that, we conducted additional research to better investigate its diagnostic and prognostic usefulness in ESCA patients. In the end, we conducted functional trials to investigate the effect that it had on the development of ESCA. Based on these findings, we hypothesized that SSTR5-AS1 could serve as an innovative biomarker for ESCA patients.

## 2. Materials and Methods

### 2.1. Cell Culture and Cell Transfections

From the American Type Culture Collection (ATCC), we got human esophageal squamous epithelial cells HET1A as well as three ESCC cell lines: EC109, KYSE150, and KYSE450. At a temperature of 37 degrees Celsius, the cells were kept alive in a medium consisting of RPMI-1640 that had been supplemented with 10% fetal bovine serum (FBS; Thermo Fisher Scientific, Waltham, USA). The siRNAs for SSTR5-AS1 and scrambled siRNA were purchased from Ribobio (Guangzhou, China). Using the Lipofectamine 2000 reagent (Invitrogen, Carlsbad, USA), siRNAs were introduced into the cells to be transfected.

### 2.2. RNA Extraction and Quantitative Real-Time PCR

The total RNA from the cultivated cells was extracted with Trizol reagent (Invitrogen, California, USA) in accordance with the instructions provided by the manufacturer. In the second step, 2 *μ*g of total RNA was subjected to reverse transcription utilizing a PrimeScript™ RT reagent kit with gDNA Eraser (Takara, Dalian, Niaoning, China) in an overall volume of 20 L in accordance with the procedure provided by the manufacturer. After that, a real-time quantitative polymerase chain reaction (qRT-PCR) was performed with the SYBR Premix Ex TaqTM II PCR Kit (Takara, Otsu, Shiga, Japan) by carefully adhering to the instrument that was provided by the manufacturer. Results were normalized to the content of GAPDH. The results of each experiment were recorded three times. The 2^−*ΔΔ*Ct^ method was used to represent the fold-change values of lncRNA expression. Primers used for RT-RCR are presented as follows: SSTR5-AS1-F: 5′-ACTACAGGTGCCATCAGACC-3′, SSTR5-AS1-R: 5′-AGCCTGCCATCCTAACACTT-3′; GAPDH-F: F: 5′-AGGTGAAGGTCGGAGTCAACG-3′, GAPDH-R: R: 5′-AGGGGTCATTGATGGCAACA-3′.

### 2.3. Cell Proliferation

For the purpose of analyzing cell proliferation, a Cell Counting Kit-8 (CCK-8, Beyotime in Shanghai, China) was applied. In a nutshell, following transfection for a period of 48 hours, various types of cells were seeded into 96-well plates. Incubation of the cells in CCK-8 solution took place for one hour on days 1, 2, and 3. After that, the optical density (OD) was measured using a microplate reader set to 450 nm. The experiments were repeated three times.

### 2.4. Patients and Datasets

The gene expression data of 182 ESCA samples from TCGA and 653 normal specimens from GTEx were derived from UCSC Xena (https://xena.ucsc.edu/). In order to provide a more accurate depiction, the expression levels of lncRNAs that were used in TCGA and GTEx were normalized to log2 (TPM+0.001).

### 2.5. Statistical Analysis

Data analysis was performed by using GraphPad Prism 6.0 (GraphPad Software, La Jolla, USA). Student's paired two-tailed *t*-test was utilized in order to analyze the statistical differences that existed between the groups. The chi-squared test was utilized in order to investigate and analyze the relationships that existed between clinical features and SSTR5-AS1 expression. The Kaplan-Meier methods were applied to plot survival curves. The Cox regression model was utilized in order to investigate the relevance of each variable's prognostic impact on overall survival. A statistically significant difference was determined to exist when *p* was less than 0.05.

## 3. Results

### 3.1. SSTR5-AS1 Was Upregulated in ESCA Patients

**Figure 1:**
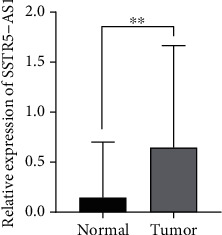
SSTR5-AS1 expression in ESCA samples and nontumor samples from TCGA and GTEx datasets was analyzed. ^∗∗^*p* < 0.01.

The observation that numerous malignancies exhibited aberrant expressions of lncRNAs has led researchers to hypothesize that these molecules are crucial regulators in the progression of tumors [26, 27]. RT-PCR was utilized to investigate the levels of SSTR5-AS1 in a total of 182 ESCA samples and 653 nontumor samples. SSTR5-AS1 exhibited a significantly increased expression in ESCA specimens vs. corresponding normal specimens (*p* < 0.01, Figure 1).

### 3.2. SSTR5-AS1 Has a Diagnostic Value for ESCA Patients

**Figure 2:**
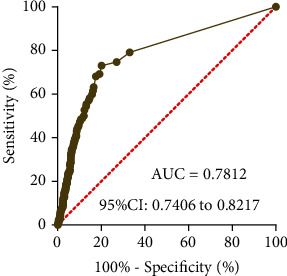
The receiver operating characteristic (ROC) curve was used to assess the diagnostic value of SSTR5-AS1 in ESCA patients.

Subsequently, the diagnostic values of SSTR5-AS1 for ESCA patients were explored. According to the ROC assays, high SSTR5-AS1 expression had an AUC value of 0.7812 (95% CI: 0.7406 to 0.8217) for ESCA (Figure 2). Our findings revealed that SSTR5-AS1 may be an indicator of the diagnosis of ESCA patients.

### 3.3. The Association between SSTR5-AS1 Expression and Clinicopathologic Features of ESCA

After that, an investigation into the connection between the amount of SSTR5-AS1 expression and the clinicopathological aspects was carried out. The average expression levels of SSTR5-AS1 were taken into consideration in order to classify ESCA patients into two distinct groups: the group of patients whose expression levels were high and the group of patients whose expression levels were low. The relations are outlined in Table 1. There was no clear connection between the expression of SSTR5-AS1 and a number of clinicopathological features (all *p* > 0.05).

### 3.4. Survival Analysis and Prognostic Significance of SSTR5-AS1 Expression in ESCA Patients

**Figure 3:**
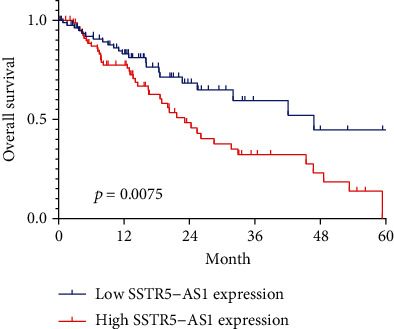
Kaplan-Meier survival curves of patients with ESCA based on SSTR5-AS1 expression.

To further investigate the correlations of SSTR5-AS1 expression level with survivals of patients with ESCA, Kaplan-Meier analyses were performed. We found that, for ESCA patients whose SSTR5-AS1 expression level was higher, the overall survival was obviously shorter than those with low SSTR5-AS1 expression level (Figure 3). For further exploring the prognostic value exhibited by SSTR5-AS1 expression in ESCA patients, a univariate Cox model was performed. As presented in Table 2, we observed that pathologic stage and SSTR5-AS1 expression were prognostic predictors for ESCA patients (all *p* < 0.05). In multivariate assays, SSTR5-AS1 expression could independently predict the clinical outcomes of SSTR5-AS1 regarding the overall survival (HR = 2.286, 95% CI: 1.268-4.122; *p* = 0.006) of ESCA patients.

### 3.5. SSTR5-AS1 Promoted ESCA Cell Proliferation

**Figure 4:**
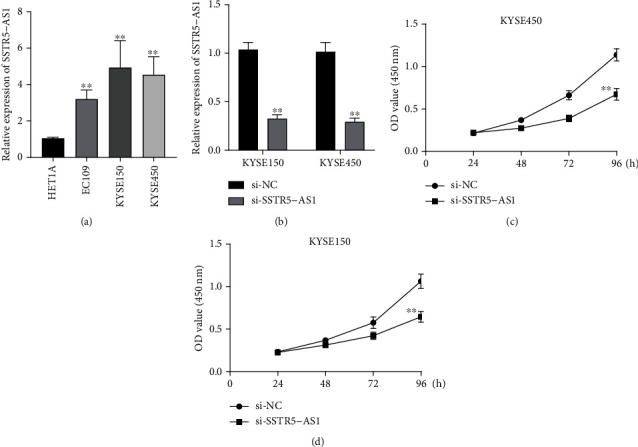
Knockdown of SSTR5-AS1 suppressed the proliferation of ESCA cells. (a) RT-PCR of SSTR5-AS1 expressions in HET1A, KYSE450, KYSE150 and EC109 cells. (b) qRT-PCR evaluation of SSTR5-AS1 expressions in KYSE450 and KYSE150 cells after the transfection. (c, d) CCK-8 assays examined cell proliferative abilities of KYSE450 and KYSE150 cells.

In order to provide evidence of the presence of SSTR5-AS1 in ESCA samples, we carried out RT-PCR and discovered that the levels of SSTR5-AS1 were noticeably higher in three ESCA cells when compared with HET1A cells (Figure 4(a)). RNA interference was used to successfully knock down the expression of SSTR5-AS1 in the KYSE150 and KYSE450 cells.  Figure 4(b) shows that SSTR5-AS1 was successfully knocked down in KYSE150 and KYSE450 cells by using the si-SSTR5-AS1 reagent (Figure 4(b)). We undertook CCK-8 tests to further investigate whether SSTR5-AS1 could play a functional role in the evolution of ESCA. The data revealed that silence of SSTR5-AS1 markedly inhibited the proliferation of ESCA cells (Figures 4(c) and 4(d)).

## 4. Discussion

The overall mortality rate for ESCA can reach as high as 88%, making it one of the most deadly forms of cancers [28]. Despite the fact that there have been some improvements made to clinical outcomes as a result of developments in treatments, the survival rate is still very low [29, 30]. There are a great number of biomarkers that have been discovered to have a correlation with survival, and there is mounting evidence to suggest that gene markers are the most accurate technique to forecast clinical outcomes [31, 32]. Thus, there is an immediate need to examine the gene expression profile of ESCA for the better examination of the outcomes of ESCA patients. This progress can be accomplished by conducting research on ESCA patients. It is possible that in the not-too-distant future, the development and validation of prognostic gene biomarkers will result in improved clinical outcomes for these individuals.

Over the course of the last few years, a significant portion of the repertoire of nonprotein-coding transcripts found in the genome, including lncRNAs, has been regarded as irrelevant “junk” associated with transcription [33, 34]. lncRNAs have recently come to the forefront of attention as a result of the completion of ENCODE and the launch of the TCGA program, which have brought to light the significant roles that they play in the development and progression of cancer [22, 35]. In individuals suffering from ESCA, the diagnostic and prognostic usefulness of lncRNAs has been the subject of a number of researches in recent years. For instance, Liu et al. showed that the expressions of LINC00963 were dramatically elevated in ESCC tissues, and its elevation was found to be linked with advanced TNM stage, metastasis, and a poor prognosis. Through the targeting of the miR-214-5p/RAB14 axis, the suppression of LINC00963 expression was able to inhibit the proliferation and invasion of ESCC cells in vitro, as well as the formation of tumors in vivo [36]. Xie et al. found that lncRNA RMRP showed considerable upregulation in ESCC, which was related with the presence or absence of lymph node metastases, the TNM stage of patients, and a poor prognosis for ESCC patients. In addition to this, RMRP regulated miR-613/NRP2, which in turn led to the promotion of ESCC cell proliferation, migration, and invasion. Through its role in the regulation of the miR-613/NRP2 axis, RMRP played a role in the progression of ESCC [37]. This finding identifies RMRP as a possible therapeutic target in the treatment of ESCC. These findings brought to light the possibility of using lncRNAs as new biomarkers for patients with ESCA.

In this study, we identified a novel ESCA-related lncRNA SSTR5-AS1 which was highly expressed in ESCA patients. In the past, a number of researches came to the conclusion that SSTR5-AS1 played a role in the development of a number of cancers. For instance, Xue et al. reported that higher SSTR5-AS1 expression was connected with a lower overall survival rate in gallbladder carcinoma patients. SSTR5-AS1 was dramatically enhanced in both gallbladder carcinoma samples and cell lines, particularly in gemcitabine-resistant cell lines. In terms of their functionality, knockdowns of SSTR5-AS1 made drug-resistant gallbladder cancer cells more sensitive to gemcitabine in vitro, and they greatly reduced the formation of xenografts in vivo by drug-resistant gallbladder carcinoma cells by stabilizing NONO [38]. Xu et al. showed that the role of SSTR5-AS1 as a ceRNA is to regulate CA2 expression by sponging miR-15b-5p, which is important for the progression and prognosis of hepatocellular carcinoma caused by HBV [39]. In addition, it was found that high expression of SSTR5-AS1 was connected to the clinical outcome of both laryngeal squamous cell carcinoma and gastric cancer [40, 41]. The clinical importance of SSTR5-AS1 in ESCA, on the other hand, has not been studied. In this study, we confirmed the diagnostic value based on the results of the ROC assays with an AUC value of 0.7812 for ESCA. Moreover, we found that patients with high SSTR5-AS1 expressions were related to poor outcomes of ESCA patients. In a multivariate Cox model, SSTR5-AS1 expression could independently predict the outcomes of SSTR5-AS1 regarding the overall survival of ESCA patients. Our findings firstly provided evidences that SSTR5-AS1 may be a functional regulator in ESCA patients and may be used as a novel biomarker. In order to provide more evidence of our findings, we performed an RT-PCR experiment. The data showed that the level of expression of SSTR5-AS1 was noticeably elevated in ESCA cells in contrast to HET1A cells. We were able to show that inhibiting SSTR5-AS1 functioned to limit the proliferation of ESCA cells, which led us to hypothesize that it may play a role as a tumor promotor in the advancement of ESCA.

There are some limitations in our study. Firstly, the sample size is relatively small, large clinical trials are needed to conduct. Secondly, the specific function of SSTR5-AS1 was not explored in vivo. In the future, we will design more complex experiments to further understand the function of SSTR5-AS1 in ESCA progression.

## 5. Conclusion

To our knowledge, this is the first study to demonstrate a close association between high SSTR5-AS1 expression and a bad prognosis in ESCA patients, suggesting that SSTR5-AS1 may serve as a useful novel molecular marker for predicting ESCA prognosis.

## Figures and Tables

**Table 1:** The association between SSTR5-AS1 expression and characteristics of patients suffering from ESCA.

Clinicopathological features	No. of cases	SSTR5-AS1 expression		*p* value
		High	Low	
Gender				0.653
Male	23	10	13	
Female	139	71	68	
Age				0.209
≤60	93	46	37	
>60	79	35	44	
Histologic grade				0.061
G1	16	13	3	
G2	66	32	34	
G3	44	24	20	
Pathologic stage				0.321
Stage I	16	6	10	
Stage II	69	40	29	
Stage III	49	23	26	
Stage IV	8	3	5	

**Table 2:** Univariate and multivariate analyses of prognostic variables of overall survival in ESCA patients.

Characteristics	Total (*N*)	Univariate analysis		Multivariate analysis	
		HR (95% CI)	*p* value	HR (95% CI)	*p* value
Age	162				
≤60	83	Reference			
>60	79	0.831 (0.506-1.365)	0.466		
Gender	162				
Female	23	Reference			
Male	139	2.306 (0.922-5.770)	0.074	1.571 (0.543-4.543)	0.405
Pathologic stage	142				
Stage I & Stage II	85	Reference			
Stage III & Stage IV	57	3.223 (1.807-5.747)	<0.001	2.961 (1.623-5.402)	<0.001
SSTR5-AS1	162				
Low	81	Reference			
High	81	2.002 (1.192-3.361)	0.009	2.286 (1.268-4.122)	0.006

## Data Availability

The analyzed datasets generated during the study are available from the corresponding author on reasonable request.
